# Developments in the Diagnostic Techniques of Infectious Diseases: Rural and Urban Prospective

**DOI:** 10.4236/aid.2018.83012

**Published:** 2018-08-23

**Authors:** Shweta Srivastava, Prabhat K. Singh, Vatsalya Vatsalya, Robert C. Karch

**Affiliations:** 1 University of Louisville School of Medicine, Louisville, USA; 2 College of Arts and Sciences American University, Washington DC, USA; 3 Maghad University, Patna, India

**Keywords:** Infectious Diseases, Microbial Disease, Diagnostic Techniques, Bacterial, Parasitic, Viral, Global Health

## Abstract

**Objectives:**

Diagnostics is the first step for the treatment and eradication of infectious microbial diseases. Due to ever evolving pathogens and emerging new diseases, there is an urgent need to identify suitable diagnostic techniques for better management of each disease. The success rate of specific diagnostic technique in any population depends on various factors including type of the microbial pathogen, availability of resources, technical expertise, disease severity and degree of epidemic of disease in the area. One of the important tasks of the policy makers is to identify and implement suitable diagnostic techniques for specific regions based on their specific requirements. In this review we have discussed various techniques available in the literature and their suitability for the target population based on above mentioned criteria.

**Methods:**

Diagnostic techniques evaluation of well documented representative microbial diseases; Tuberculosis (bacterial), Malaria (parasitic) and HIV (viral) were included in the study. Identification and collection of information and data was performed focusing on the diagnostic techniques used from the scientific publications from Pubmed, Science Access, Scopus, EMBASE and several regional databases. WHO and CDC database for Tuberculosis, Malaria and HIV were also included. These techniques were compared with respect to the financial resource availability, expertise and management, functional capacity, pathogen virulence and degree of epidemic in the population.

**Results and Conclusion:**

In case of Tuberculosis, ELISA and colorimetric techniques are successful in rural and urban communities with 80% – 90% sensitivity. Genotyping and SNP analysis are useful in drug resistant strains. Parasitic disease Malaria also follows the same trend with diagnostic techniques like RDTs being common in both population with fast results and around 90% sensitivity. STD disease like HIV however shows slight different trends due to urgent need of interference in rural epidemics of the disease. Rapid and sensitive immunotechniques like dipsticks and agglutination with almost 100% sensitivity are used in both rural and urban areas. For the confirmation further tests are done like protein Western and NAAT. Advance techniques could be the option for higher epidemic area, drug resistance and disease research, while rapid techniques would be suitable for low income areas and POC facilities. Therefore, suitability of the diagnostic techniques for better management depends not only on the financial resources and assessment skills of a community but sometimes on the disease itself. We have further discussed the technological improvements for specific settings (rural/urban) based on the past research for better management of diseases, which could be implemented for the understanding of understudied and newly emerging diseases.

## 1. Introduction

Infectious diseases are the leading cause of morbidity and mortality across the world. Correct and timely diagnosis is the first step on the path to treatment as well as disease control and prevention. Effective diagnostic techniques are important for the disease identification and proper treatment as well as control of outbreaks in the population. Whether these techniques are valuable in given community setting and if so, then which test could be most appropriate; are some important concerns that can be answered through evaluations of these techniques with respect to many factors [1] [2]. To address the tropical disease diagnostics and treatment in the developing countries, WHO (World Health Organization) along with UNICEF (United Nations Children’s Fund), world bank and UNDP (United Nations Development Program) have come up with a special program TDR (http://www.who.int/tdr/en/) who arranged an expert advisory panel for designing and conducting of standard diagnostic evaluation. Another collaboration of WHO with FIND (https://www.finddx.org/) is working on policy making and implementation for testing and providing effective diagnostic techniques of infectious diseases to different countries based on their specific requirements. In the era of Infectious disease epidemics and emerging new diseases, there is need for identification of effective and readily available diagnostic techniques and timely management for treatment. Availability and access of resources, expertise in current technique that could add understanding of the virulence, genetic variation of the pathogen, and severity of the disease are important factors. In the literature, various diagnostic methods for the infectious diseases have been proposed and tested. Main stream diagnostics can be classified into three broad categories: 1) classical methods, like microscopy [3] and cell culture [4]; 2) biochemical methods, like immunoassays [5] [6] and colorimetric test [7] and [8]; 3) advance biotechnology methods like molecular genotyping [9] [10] [11] [12] [13], DNA microarray [14] [15], and nanotechnology [16]. Each of the methods has their own advantages and limitations within their range of functions and the circumstances, in which they are required and performed. Classical methods are considered to be gold standards and cost effective, while advance methods are faster and more sensitive in many cases. Classical methods like microscopy and culture are well established and affordable methods for certain microbial pathogens like Tuberculosis [3]. These methods are easily accessible in hard to reach rural area compare to expensive modern techniques. Despite these advantages, the gold-standard diagnostic methods have limitations, including laborious sample preparation, slow results, less sensitivity and sometimes as ineffective detection. In present perspective, with regions-specific requirements, where new diseases and pathogens are emerging every day, more accurate and rapid techniques are requirement. Considering these characteristics, researchers have utilized innovative approaches of biotechnological methods. Rapid molecular methods have enhanced the capabilities of laboratories to identify and characterize microbial pathogens in detail [17]. However, given the limited resources available, especially in developing countries, the new techniques should be prioritized for correct policy decisions. Focus of this review is to evaluate and identify better diagnostic techniques based on literature survey available for certain significant infectious diseases. Majority of the microbial infectious disease are caused by bacteria, parasite or virus. Therefore, representative diseases selected for this review are widely studied Tuberculosis (bacterial) Malaria (parasite) and AIDS (virus). Another objective of this study is to provide information for diagnostic implementation in context of rural and urban communities as well as burden and severity of the disease.

## 2. Methods

Identification and collection of information and data was performed focusing on the diagnostic techniques used from the scientific publications on or before August 2017 in English language from Pubmed, Science Access, Scopus, EMBASE and were searched. WHO and CDC database for Tuberculosis, Malaria and HIV were also included in the study. We searched the reports of primary clinical, epidemiological and laboratory studies about diagnostic developments and its efficacy in the light of specific microbial disease. Some of the keywords for the search were: Microbial AND diagnostic techniques, Tuberculosis/Malaria/HIV AND Diagnostic techniques, Diagnostic methods AND infectious disease, Africa AND HIV, Southeast Asia AND microbial diagnostic techniques etc. Target populations for the study were underdeveloped communities in the countries of Africa and Southeast Asia as well as developed European and American communities. References were selected on the basis of efficacy of the technique studied, size of the population studied, success of the studied technique in relevant population, techniques recommended by WHO/CDC. These techniques were compared with respect to the financial resource availability, expertise and management, functional capacity, and degree of infection.

## 3. Review of Techniques

### 3.1. Diagnostic Techniques for Tuberculosis

Tuberculosis (TB) is an airborne contagious bacterial disease, which ranks as the second leading cause of death from an infectious disease worldwide, after HIV. According to the 2012 World Health Organization [18] global TB report (http://apps.who.int/iris/bitstream/10665/75938/1/9789241564502_eng.pdf), in the year 2011 itself 8.7 million people fell ill with TB while 1.4 million died due to the disease [19]. Lack of adequate diagnostic measures for timely detection were the main concern preventing proper response to tackle the morbidity and mortality, especially in the HIV associated and drug resistant TB cases. In low income and high incidence countries, diagnosis is still dependent on traditional techniques such as sputum smear light microscopy and sometimes culture. However, these microscopy techniques are far less sensitive ranging from 20 to 80 percent sensitivity [20] especially in HIV patients and children where pulmonary bacillary load is less than detection limits of microscopy [21]. To improve sensitivity, WHO has recommended use of light emitting diode [22] microscopy which can generate both light and fluorescence wavelength instead of conventional light or fluorescence microscopes [23]. Little Improvement in microscopy technique was still not appropriate to tackle diagnostic challenges such as HIV co-infected patients and drug resistance cases, which made cultivation indispensable. According to the WHO guidelines, microscopy negative HIV patients with TB symptoms are to be tested by culture as well (Table 1).

Multidrug resistant TB, broadly categorize as MDR-TB (mainly resistant to INH and RIF) and XDR-TB (Resistant to additional antibiotics) are the major concerns for the need of rapid and effective diagnostic techniques which is traditionally identified by conventional culture and drug susceptibility test (DST). Culture techniques in the resource poor countries are inefficient due to lack of infrastructure, poor biosafety measures as well as unavailability of trained staff to perform reliable tests. Moreover, crucial time is lost during cultivation and DST. Various immunological techniques like serologic test and Enzyme-linked immunosorbent assay (ELISA) were also tested for the TB diagnosis but were not successful due to low sensitivity, and cross reactivity [24] [25]. More advance techniques like DNA based molecular line probe assays [26] have been introduced based on the genetic studies suggesting that the drug resistance in certain strains is due to mutation at the drug target site [27] [28] [29]. Line probe assays [26] is a Polymerase chain reaction (PCR) based reverse hybridization molecular drug susceptibility assay which is very specific (>99%), sensitive (>97%) and rapid and does not require viable pathogen for the detection which makes handling and biosafety more convenient. However, LPA probes are mainly specific for MDR-TB but not extensively drug-resistant tuberculosis (XDR-TB) since no single mutation is responsible for extensive drug resistance [29] [30]. Therefore, advent of LPA did not eliminate the need of conventional cultivation especially for the diagnosis of XDR-TB. In 2009 WHO endorsed LPA coupled with liquid media cultivation technique for TB diagnosis in endemic countries [23] (Table 1).

Real time PCR (RTPCR) is one of the advance and rapid DNA based method, which amplifies DNA in a closed system and gives DNA melting profiles to detect resistance associated mutation. One of the fully automated real time PCR based technique named Xpert MTB/RIF can detect TB, and identify rifampicin resistance directly from sputum under two hour [31]. Clinical validation of the Xpert MTB/RIF technique suggested 100% specificity for smear positive culture positive as well culture negative cases [31]. Xpert MTB/RIF system has offered excellent detection performance with lower biosafety requirements and ease of equipment operation. Compact real time PCR Xpert MTB/RIF system is easy to transport and thus can provide onsite diagnosis at point of care to the patients. The major limitation of the Xpert MTB/RIF method is the high cost of reagents and instrument compare to LPA or other assays. Since majority of the drug-resistance cases are rifamycin therefore WHO has endorsed the Xpert technology in 2010, and is monitoring the global roll out of the technology to promote effective coordination [32]. The TB-Xpert Project will provide approximately 1.4 million Xpert MTB/RIF test cartridges and over 200 GeneXpert instruments for the rapid detection of TB and rifampicin resistance in 21 South East Asian and African endemic countries from year 2013 to 2015 [19]. However, Xpert MTB/RIF technology does not eliminate the need for conventional microscopy culture and DST, which are required to monitor treatment progress and to detect resistance to drugs other than rifampicin. In settings or patient groups where rifampicin resistance is rare, Xpert MTB/RIF results indicating rifampicin resistance should be confirmed by conventional DST or LPA. One of the most promising and upcoming diagnostic technique is the DNA microarray chip platform which can detects all the gene mutations simultaneously to target any drug resistance [33] [34] (Table 1). Microarray technique can perform identification, genotyping as well as drug resistance due to every known mutation in one experiment simultaneously. Equipped with immense potential; microarray technique is still at the stage of infancy and would require lots of optimization and clinical trials before it becomes a standard diagnostic technique for TB.

Looking at the overall scenario and present challenges no single technique is the gold standard for TB diagnosis. Therefore, an integrated tiered level approach is advisable where the diagnosis of the disease is performed in the laboratories at different levels. Each level is divided based on the complexity and availability of the resource and trained personnel. The very first level should be onsite or can be in the rural area where simple microscopy and sample collection can be performed. Next level should be the laboratories where samples can be transferred to perform better microscopy, conventional culture and DST tests with adequate measures. Final level should be sophisticated hi-tech laboratories headquarters, which can perform genotyping, further drug resistance test and research for better diagnosis and cure. Coordination between each level of the laboratories is the most important step towards successful management of the disease.

### 3.2. Diagnostic Techniques for Malaria

Malaria is one of the most prevalent and deadly parasitic diseases especially in the underdeveloped countries of Africa and South East Asia. According to the latest report from WHO there were about 219 million estimated cases of malaria in 2010 (with an uncertainty range of 154 million to 289 million) and 660,000 deaths (with an uncertainty range of 490,000 to 836,000) [18]. In the year 2012, new initiative from WHO global program T3 (Test, Treat and Track) developed to provide universal access to diagnostics, treatment and stronger surveillance. Early diagnosis is important for the proper treatment, and control of transmission of disease. Most evaluated and successful techniques for Malaria diagnosis so far are Giemsa microscopy and rapid diagnostic tests (RDTs) [35] [36]. Due to its lower cost and simplicity, giemsa staining microscopy still remains the standard method for rapid detection of parasite in rural endemic area [37] (Table 2). However, Low sensitivity (50 – 100 parasite per μl), false positive results and emerging complications in the diagnosis like dealing with 4-aminoquinolines drug resistance P. falciparum strain and low level of infection make conventional techniques inadequate for the purpose. In the last few decades of malaria research alternative methods like ELISA [38], Immunofluorescence assay [39], RDTs [40] [41] and recently DNA based assays have been introduced. Among them, so far RDTs have shown promising results due to its similar sensitivity to microscopy (200 parasites per μl in clinical settings) but ease of use with no instrumentation or technical skill requirement and point of care (POC) availability. In 2006, WHO, Special Program for Research and Training in Tropical Diseases [42] and the Foundation for Innovative New Diagnostics (FIND) launched an evaluation program to assess the comparative performance of commercially available malaria RDTs. SO far four rounds of testing have been performed on 164 RDT products and published [36]. P. falciparum tests targeting HRP2 antigen demonstrated the highest PDS however tests targeting pLDH for P. falciparum and P. vivax detection did not pass round 1 (<80% PDS for P. falciparum at 2000 parasites/μl). The results of the worldwide RDT evaluation program would further guide policy makers of government agencies towards deciding better-performing tests.

Non-sensitivity for all Plasmodium species, thermosensitivity, inability to detect low level of infection (less than 200 parasites per μl), and false positive results are the major concerns for RDTs at this point for efficacy of these standard diagnostic measure. DNA based diagnostic techniques have advantage of being more sensitive, specific, determining species, drug resistance and low level of infection. PCR is one of the basic and sensitive DNA based technique and has limit of detection up to 0.5 – 5 parasites/ml [43] [44]. Isothermal amplification methods such as Loop mediated isothermal amplification (LAMP) is widely studied DNA based amplification technique in Malaria diagnosis, which does not require thermos-cycler and has 95% sensitivity, and 99% specificity with documented detection limit of 0.2 parasite/ml [45] [46]. Other DNA based techniques such as real-time PCR, Multiplex PCR/Ligation Detection Reaction (PCR-LDR), and Ligase Detection Reaction-Fluorescent Microsphere Assay (LDR-FMA) have also been introduced and tested (Table 2). Major drawback of the DNA amplification techniques are expensive reagents, instrument requirements and special care in handling of samples as they are prone to contamination and amplification of non-targeted DNA sequences. Currently, these techniques are limited to high profile lab or central health care facilities due to their resource intense requirements and high cost. Novel strategies are needed to further research to improve and incorporate these techniques into routine health centers in endemic areas.

Overall, in the present scenario both low technology and high technology approaches are indispensable for successful parasite detection towards management and eventually in the eradication of the disease. RDTs and microscopy are suitable for the majority of symptomatic P. falciparum detection and management while molecular based advance techniques are required for detection of low level of infection and asymptomatic individuals who may contribute to continuing malaria transmission and P. *vivax* cases.

### 3.3. Diagnostic Techniques for AIDS

AIDS, caused by HIV is the major public health issue in the world. According to a recent WHO and UNAIDS data, 36.9 million people were living with HIV globally at the end of 2014 while 1.2 million people died and 2 million newly infected [47]. Sub-Saharan Africa is the most affected region accounting for almost 70% of global HIV infection. There is no cure for HIV. However, timely detection the HIV status can be beneficial for effective antiretroviral therapy (ART) for productive lifestyle and preventing the spread of the disease. In present days, there are three types of popular HIV diagnostic tests available including antibody tests like ELISA, rapid test or Western blot [48], antigen/antibody combination tests like viral protein p24 along with HIV antibody [49] [50] [51], and nucleic acid tests (NAT) [52] [53] (Table 3). Antibody tests detect antibodies, proteins that the body makes against HIV, not HIV itself. Antigen tests and RNA tests detect HIV directly. Fourth generation techniques for detection of antibody and antigen simultaneously can reduce the time of diagnostic window after primary infection compared to antibody alone [54]. Nucleic acid amplification test (NAAT) mainly rely on amplification of the nucleic acid by PCR and can be qualitative as well as quantitative. PCR assays have become more popular nowadays due to its sensitivity and ease of technique [55]. Since the advent of human immunodeficiency virus [26] testing, laboratory based methods have undergone tremendous change. Western blot and indirect immunofluorescence assay [56] have been excluded in the updated CDC recommendations due to false negative results [57] [58] [59]. An initial HIV antibody test or antigen/antibody test is performed along with some more follow-up confirmatory testing as per the updated Centers for Disease Control and prevention [36] and WHO guidelines [60]. Based on updated CDC guidelines (Laboratory Testing for the Diagnosis of HIV Infection: Updated Recommendations Published June 27, 2014, https://stacks.cdc.gov/view/cdc/23447), laboratory personnel should use Food and Drug Administration (FDA) approved assays for the diagnosis of HIV infection in adults and children > 24 months of age. Testing should be performed with ag/ab detection tests, a combination immunoassay that detects HIV1 and HIV2 antibodies. All positive specimens on this initial assay should undergo further testing with an immunoassay that differentiates HIV-1 from HIV-2 antibodies. Specimens that are reactive on the initial immunoassay and non-reactive or indeterminate on the antibody differentiation assay proceed to HIV-1 nucleic acid testing for resolution, which looks for the virus RNA directly. Positive results from the recommended algorithm indicate the need for HIV medical care, and an initial evaluation that includes additional laboratory tests (such as HIV-1 viral load, soluble cells of differentiation [sCD4+], T lymphocyte determination, and antiretroviral resistance assay) to confirm the presence of HIV-1 infection. It is used further to stage HIV disease, and to assist in the selection of an initial antiretroviral drug regimen (OARAC, Panel on Antiretroviral Guidelines for Adults and Adolescents). Guidelines for the use of antiretroviral agents in HIV1-infected adults and adolescents is available electronically at (http://www.aidsinfo.nih.gov/ContentFiles/AdultandAdolescentGL.pdf last updated October 2017. In the 2012 Geneva meeting, WHO has recommended multitier approach to diagnose and treat HIV in epidemic and non-epidemic areas especially in the developing countries based on the resource availability (WHO Expert Meeting Report Geneva, Switzerland, 6 – 7 June 2012: http://apps.who.int/iris/bitstream/10665/75971/1/9789241504522_eng.pdf).

Level 0: Community outreach setting: Community health worker for spreading awareness, HIV RDTs (Rapid diagnostic tests).

Level 1: Primary care setting: trained health care workers: nurses, clinical officers HIV RDTs, other POC tests, database collection.

Level 2: District: Laboratory technicians and assistants EIA for diagnosis, low throughput soluble CD4 (sCD4+), chemistry, hematology, microbiology.

Level 3: Regional or provincial: Laboratory specialists/senior technicians EIA for diagnosis, higher throughput sCD4, HIV molecular technologies including HIV VL, quantitative/qualitative “Early infancy detection” (EID).

Level 4: National: Senior laboratory specialists using enzyme immunoassays (EIA) for diagnosis, higher throughput sCD4, HIV molecular technologies including HIV viral load (VL), quantitative/qualitative EID, HIV resistance testing.

## 4. Conclusion

The techniques evaluated for the diagnostics in population were classical like microscopy, immunoassays like ELISA and colorimetric assay and advance biotechnological methods like genotyping. In case of bacterial diseases like Tuberculosis ELISA and colorimetric techniques are common in rural and urban communities with 80% – 90% sensitivity. Microscopy and cultivation though common but has low sensitivity and cultivation requires specific media and time taking. Genotyping and SNP analysis are mainly performed in urban labs due to their sophistication are not only 100% sensitive but also useful in drug resistant strains study. Parasitic disease Malaria also follows same trend with diagnostic techniques like immunoassay and RDTs based on immunoassay being common in both rural and urban population with fast results and around 90% sensitivity. High throughput genotyping methods however at this time are limited to urban labs and are useful for studying new emerging and resistant strains. STD disease like HIV however shows slight different trends in terms of diagnostic development due to urgent need of interference in rural epidemics of the disease. We now have rapid and sensitive immunotechniques available like dipsticks and agglutination, which can determine with almost 100% sensitivity positivity or negativity and used in both rural and urban areas. For the confirmation further tests are done like protein Western etc. More sophisticated RNA NAAT test used in urban lab is more advance and sophisticated not only for early detection of infection, but also to determine the load of infection. Our preliminary observation suggests that advance biotech techniques may be the option for developed countries, while cheap, effective and less complicated techniques would be suitable for low income developing countries. However rapid and innovative techniques are necessary in case of highly infectious and severe disease for timely management. Therefore, suitability of the diagnostic techniques for better management depends not only on the financial resources and assessment skills of a community but sometimes on the disease itself.

## Figures and Tables

**Table 1.** Tuberculosis diagnostic techniques studied on different populations and WHO/CDC recommendations.

Diagnostic technique	Efficacy in Rural/Urban	Advantage	Limitation	Reference	WHO/CDC recommendation
Light Microscopy of sputum smear	Rural/Urban	Low cost, easy accessible traditional method.	Low sensitivity. Not suitable for drug resistant strains	[3] [61].	Discontinued by WHO.
Fluorescence microscopy	Rural/Urban	One of the traditional method.	High setting and cost. Not much difference in sensitivity from light microscopy	[62] [63] [64] [65].	Discontinued by WHO.
LED fluorescence microscopy	Rural/Urban	Higher sensitivity compared to traditional microscopy. Useful in peripheral area for detection.	The low sensitivity HIV-positive individuals particularly those with low CD4 T cell counts. Not suitable for drug resistant test.	[66] [67] [68].	WHO has recommended use of LED microscopy which can generate both light and fluorescence wavelength instead of conventional light or fluorescence microscopes [55].
Culture and Drug susceptibility test	Urban lab settings with biosafety level 3 lab (BSL3) requirements	More sensitive than microscopy. Drug resistance can be confirmed.	Time taking, expensive, Lab setting and expertise needed. Risk of cross contamination and biohazard.	[3] [69] [70].	Commercial liquid culture medium and rapid speciation strip recommended by WHO [23] [71].
Immunological technique-(ELISA /RDTs)	Quick serodiagnostc tests in rural and urban settings	Quick commercial tests, ease to use.	Low sensitivity, False positive results	[5] [72].	Recommended to discontinue commercial serodiagnostic tests by WHO [18].
DNA based test-LPA	Commercial kits available for rural and urban setting with biosafety level 2 lab (BSL2) requirement	High sensitivity test. Less sample requirement. Drug resistance can be detected and correlated to gene mutation.	Mainly recommended for MDR-TB but not for XDR-TB.	[27] [28] [29] [73].	The use of commercial line probe assays is recommended by WHO in MDR-TB endemic area as well as combination with cultivation for DST [71].
DNA based test-RTPCR	Fast commercial Xpert method and GenXpert instruments for rural and urban area	rtPCR based technique Xpert MTB/RIF can detect and identify drug resistance directly from sputum. Highly sensitive, cost effective, less time taking, drug resistance detection, lower biosafety requirements.	Only detects Rifampicin resistance	[31] [74] [75].	WHO has endorsed the Xpert technology in 2010 [19] [32].
DNA based test-Microarray technique	Urban laboratory settings	Highly sensitive and advanced method for in depth genomic studies of positive samples. Simultaneously detect all gene mutations.	Requires advanced lab settings.	[33] [34].	Not recommended for routine diagnostics.

Abbreviations: Light emitted diode [22], ELISA (enzyme-linked immunosorbent assay), Rapid detection test [26], Line Probe Assay [26], Real Time Polymerase chain reaction (RT PCR).

**Table 2.** Malaria diagnostic techniques studied on various populations and WHO/CDC recommendations.

Diagnostic technique	Efficacy in Rural/Urban	Advantage	Limitation	Reference	WHO/CDC recommendation
Microscopy Giemsa or Acridine orange staining	Rural and urban settings	First line of standard diagnostics. Cost effective.	Less sensitive. Drug resistance not detected.	[8] [37].	WHO recommends prompt diagnosis by microscopy or rapid diagnostic test (RDTs) [76] [77].
Immunological test-RDTs	Rural and urban	Ease of use. Rapid results.	Less sensitive in low parasite count. False positive/negative. Drug resistance not detected.	[35] [40] [78] [79].	WHO recommends prompt diagnosis by microscopy and commercial RDTs in endemic area [76].
Serologic test-ELISA	Rural and urban	Rapid detection. More sensitive than microscopy.	False positive, less sensitive. Lab setting needed. RTDs are better evolved immunological technique for POC.	[38] [80]	Not recommended for regular diagnostics.
Immunofluorescence assay	Urban	High sensitive than microscopy.	Requires lab settings. Not cost effective. Time taking.	[39].	Not recommended for regular diagnostics.
DNA based assay-PCR, RT PCR, Multiplex PCR/PCR-LDR, LDR-FMA, LAMP	Urban	High sensitivity and specificity. Drug resistance detection.	Standard lab settings required. Expertise needed. Not cost effective.	[43] [44] [45] [46].	Not recommended for regular diagnostics. More useful in confirmation of parasite species and drug susceptibility.

Abbreviations: Rapid detection test [26], ELISA (enzyme-linked immunosorbent assay), Ligation Detection Reaction (PCR-LDR), Ligase Detection Reaction-Fluorescent Microsphere Assay (LDR-FMA), Loop mediated isothermal amplification (LAMP).

**Table 3.** HIV/AIDS diagnostic techniques studied on different populations and WHO/CDC recommendations.

Diagnostic technique	Efficacy in Rural/Urban	Advantage	Limitation	Reference	WHO/CDC recommendation
Immunological test-Antibody test	Rural and urban	Cost effective.	Less sensitivity. False negative if ab concentration is low. Time taking.	[48].	Combination ag/ab assay recommended by WHO/CDC instead of ab alone assay (WHO 2012; CDC 2014)
Immunological test-antigen/antibody (Ag/Ab) combination assay	Rural and urban	More sensitive than antibody test alone. Faster detection window.	False positive. Specific for HIV-1/2 antigen/antibody used	[49] [50] [51] [54].	Combination ag/ab assay recommended by FDA/CDC/WHO [60] as first step in HIV detection.
NAAT	Urban	Most sensitive. Can detect and quantitate virus to stage the disease condition for therapy consideration.	High technology lab settings required. Skilled personnel need for the test. Not cost effective. Should be use to confirm and assist in therapy planning after ag/ab test.	[52] [53].	Combination ag/ab assay recommended by FDA/CDC (CDC 2014) as first step followed by further confirmation by NAAT [81].

Abbreviations: Nucleic acid amplification test (NAAT).

## Abbreviations

AIDS: Acquired Immunodeficiency Syndrome
ART: antiretroviral therapy
CDC: Centers for Disease Control and Prevention
DST: Drug Susceptibility Test
EIA: Enzyme Immunoassays
EID: Early Infancy Detection
ELISA: Enzyme-linked Immunosorbent Assay
FDA: Food and Drug Administration
FIND: Foundation for Innovative New Diagnostics
IFA: Indirect Immunofluorescence Assay
LAMP: Loop mediated isothermal amplification
LDR-FMA: Ligase Detection Reaction-Fluorescent Microsphere Assay
LED: Light Emitting Diode
LPA: Line Probe Assays
HIV: Human Immunodeficiency Virus
MDR-TB: Multi-drug-resistant Tuberculosis
PCR: Polymerase Chain Reaction
PCR-LDR: Multiplex PCR/ Ligation Detection Reaction
POC: Point of Care
RDTs: Rapid Diagnostic Tests
RIF: Rifampin
RNA: Nucleic Acid
sCD4+: Soluble Cells of Differentiation Type 4
TB: Tuberculosis
TDR: Research and Training in Tropical Diseases
VL: Viral Load
WHO: World Health Organization
XDR-TB: Extensively drug-resistant tuberculosis
